# Fluorine-Free, Highly Durable Waterproof and Breathable Fibrous Membrane with Self-Clean Performance

**DOI:** 10.3390/nano13030516

**Published:** 2023-01-27

**Authors:** Jinchao Zhao, Teng Zhang, Youmu Li, Leping Huang, Youhong Tang

**Affiliations:** 1 Hubei Provincial Engineering Laboratory for Clean Production and High Value Utilization of Bio-Based Textile Materials, Wuhan Textile University, Wuhan 430200, China; 2 School of Material Science and Engineering, Wuhan Textile University, Wuhan 430200, China; 3 Flinders Institute for NanoScale Science and Technology, College of Science and Engineering, Flinders University, Adelaide, SA 5042, Australia

**Keywords:** fibrous membrane, waterproof and breathable, superhydrophobic, self-cleaning, durability

## Abstract

Lightweight, durable waterproof and breathable membranes with multifunctional properties that mimic nature have great potential for application in high-performance textiles, efficient filtering systems and flexible electronic devices. In this work, the fluoride-free triblock copolymer poly(styrene-b-butadiene-b-styrene) (SBS) fibrous membrane with excellent elastic performance was prepared using electrospinning. According to the bionics of lotus leaves, a coarse structure was built onto the surface of the SBS fiber using dip-coating of silicon dioxide nanoparticles (SiO_2_ NPs). Polydopamine, an efficient interfacial adhesive, was introduced between the SBS fiber and SiO_2_ NPs. The hydrophobicity of the modified nanofibrous membrane was highly improved, which exhibited a super-hydrophobic surface with a water contact angle large than 160°. The modified membrane retained super-hydrophobic properties after 50 stretching cycles under 100% strains. Compared with the SBS nanofibrous membrane, the hydrostatic pressure and WVT rate of the SBS/PDA/SiO_2_ nanofibrous membrane improved simultaneously, which were 84.2 kPa and 6.4 kg·m^−2^·d^−1^ with increases of 34.7% and 56.1%, respectively. In addition, the SBS/PDA/SiO_2_ nanofibrous membrane showed outstanding self-cleaning and windproof characteristics. The high-performance fibrous membrane provides a new solution for personal protective equipment.

## 1. Introduction

Waterproof–breathable fabric can protect the human body against rain and snow in harsh environments efficiently and at the same time allow the water vapor generated by the body to transmit to the environment to maintain wearing [1,2,3,4]. Therefore, waterproof–breathable fabric is vividly called the “second skin” of the human body. It has been widely applied in functional clothing such as ski suits, surgical gowns, firefighter uniforms, army boots and spacesuits [5,6,7] and other industrial aspects of membrane distillation [8], protective coatings on buildings [9] and flexible electronic devices [10]. The performance of the waterproof–breathable fabric is determined by the function layer of the waterproof–breathable membrane. According to the intrinsic and wetting characteristics, the waterproof–breathable membrane can be classified into two categories: hydrophilic dense membranes and hydrophobic porous membranes [11]. The thermoplastic polyurethane (TPU) non-porous membrane is a typical hydrophilic dense membrane that has excellent water resistance performance (hydrostatic pressure of 140 kPa) but poor moisture permeability (water vapor transmission (WVT) rate of 2 kg·m^−2^·d^−1^) owing to its nonporous structure [12]. In contrast, the typical hydrophobic porous membrane of the polytetrafluoroethylene (PTFE) membrane shows a lower waterproof property (hydrostatic pressure of 110 kPa) but outstanding breathable performance (WVT rate of 6.3 kg·m^−2^·d^−1^) owing to its hydrophobicity and countless porous structure [13,14]. Hence, the character of pores in the membrane has always been the limit for enhancements in both waterproof and breathable performance. Although new commercial PTFE membranes claimed to have very high hydrostatic pressure above 196 kPa and WVT rate above 20 kg·m^−2^·d^−1^ [15], widely used fluorochemicals found in water, soil and people’s bodies are harmful to the environment, which limited the application of the PTFE membrane and fluorocarbon additives. Thus, an environmentally friendly waterproof–breathable membrane is urgently needed.

In order to obtain a superior porous waterproof and breathable membrane, a series of technologies including phase separation [16,17], the template-based strategy [18,19], biaxial stretching [20] and the electrospinning technology [21] have been developed. Among them, the electrospinning technology has been proven to be an effective method for fabricating porous membranes with the advantages of small pore size, high specific surface area, high porosity, interconnected tortuous channels and soft fibrous structure [22,23]. Since the polyurethane (PU)-based porous waterproof–breathable membrane was reported in 2007 [24], various polymer materials such as polyvinylidene fluoride (PVDF) [25], polyacrylonitrile (PAN) [3] and polypropylene (PP) [26] have been developed for the fabrication of waterproof–breathable membranes. Among them, PU is the most used elastomer, which gives the membrane good elasticity and deformation recovery properties [27,28]. However, the hydrophilic carbamate groups and ether groups in the main chain of PU lead to a relatively low waterproof property (5 kPa) for relevant electrospun membranes [29], which requires further modifications [30,31]. Doping with low surface energy materials proved to be an efficient method to enhance the surface hydrophobicity of the membrane [13,32]. Ge et al. fabricated a waterproof–breathable membrane by adding the per fluoroalkane segment (-C_8_F_17_) to the PU solution, which showed a hydrostatic pressure of 39.3 kPa and a WVT of 9.2 kg·m^−2^·d^−1^ [29]. Gu et al. developed a one-step electrospinning method by adding hydrophobic silica gel to the PU solution, and a waterproof–breathable membrane with a water contact angle of 142°, a hydrostatic pressure of 50.45 kPa and a WVT of 8.05 kg·m^−2^·d^−1^ was obtained [28]. However, the dopant-modified method was not suitable for all materials, and especially showed a limited effect on improving the hydrophobicity of hydrophilic polymers because of hard dispersion [33]. Furthermore, dip coating, blade coating and vapor deposition [34] methods have been developed. Among them, the dip coating method was considered the simplest and most effective method [35]. Gu et al. modified the surface of the optimized PU membrane by immersing it in a 1 wt% methanol solution of fluoroalkyl silane for 24 h, and the modified membrane exhibited a good hydrophobic surface with a water contact angle of 140° and excellent waterproof properties with a hydrostatic pressure of 126.1 kPa and a WVT rate of 9.06 kg·m^−2^·d^−1^ [27]. Zhao et al. impregnated a nynon-6 electrospun membrane in water-based alkyl acrylates and a nano TiO_2_ emulsion. The modified nynon-6 membrane demonstrated outstanding waterproof properties with a water resistance of 106.2 kPa and a moisture permeability of 10.3 kg·m^−2^·d^−1^ [36]. The polysiloxane-modified polyacrylonitrile nanofibrous membranes were prepared with the dip-coating method, which obtained a good water resistance of 93.8 kPa and a modest vapor permeability of 4.7 kg·m^−2^·d^−1^ [37].

In addition to waterproof properties and moisture permeability, a self-cleaning characteristic is also important to the waterproof and breathable membrane, to avoid performance degradation caused by stains and dust. Inspired by the bionics of the lotus leaf, a rough surface composed of micron- and nano-scale structures prevents dirt and water from adhering [38]. Nanoparticles, such as SiO_2_ and TiO_2_, are commonly used, which have been added to the spinning solution [25,39] or coated on the membrane with an aftertreatment [4,34] to create the micro–nano convex structure. The modified membranes have obtained super-hydrophobic surfaces (above 150°) and excellent self-cleaning properties [4,25,34,39]. However, hard dispersion of nano fillers in the spinning solution not only causes blockage of the nozzle but also forms random aggregation in the fiber [40], leading to performance deterioration of the membrane. Apparently, aftertreatment using coating methods can adjust the type, content, and orientation of the nanomaterials conveniently. However, the nanomaterials are detached easily from the surface of the membrane caused by friction, and the property of the membrane decreases. Thus, a binding force between the nanomaterials and the membrane is a critical factor influencing the durability of the membrane [34]. Since reported in 2007 [41], polydopamine (PDA) has been widely used as an interfacial adhesive because of its material-independent and mild reaction characteristics [42]. Meanwhile, PDA has been proven as a biocompatible and ultra-stable coating for micron- and nano-scale structures [43]. Thus, PDA is the perfect candidate for an interfacial adhesive to enhance the binding force between the nanomaterials and the membrane.

Considering resilience to mechanical deformation (e.g., stretching or compression) during frequent deformation and shape recovery is particularly important for a waterproof and breathable membrane to work on dynamic deformation [44]. The thermoplastic elastomer of the triblock copolymer poly(styrene-b-butadiene-b-styrene) (SBS) is a good option for designing a waterproof–breathable membrane, which has excellent elasticity, good corrosion resistance and easy processing characteristics, and, more importantly, it is inherently hydrophobic [45]. While benefiting from the surface chemistry and microstructure of the lotus leaf, the membrane with micro-nano convex surface demonstrates super-hydrophobic properties, self-cleaning performance and good moisture permeability [38,46]. The hydrophobic fumed silica nanoparticles (SiO_2_ NPs) can be applied to construct a “nano convex”. PDA with strong adhesion, which is introduced between the SBS fibrous membrane and SiO_2_ NPs, can adhere hydrophobic SiO_2_ NPs to the fiber surface. At the same time, hydrophilicity is another remarkable feature of PDA which can play the role of “adsorption-diffusion-desorption” in moisture permeability. So, a simple and efficient method to fabricate a fluorine-free fibrous membrane with multilevel structures by combining electrospinning and surface coating was designed. Additionally, the morphology, porous structure, surface wettability, waterproof property, vapor permeability and self-cleaning performance of the modified fibrous membrane were fully investigated here. The SBS/PDA/SiO_2_ fibrous membrane as an advanced protective material is a good candidate for personal protective equipment.

## 2. Experimental Section

### 2.1. Materials

Poly(styrene-b-butadiene-b-styrene) (SBS, YH-791H, M_w_ = 100,000, S/B ratio: 30/70) was obtained through Yueyang Baling Huaxing Petrochemical Co., Ltd., Yueyang, China. N, N-dimethylformamide (DMF, AR, ≥99.5%), Tetrahydrofuran (THF, AR, ≥99.5%), 1M Tris-HCl ((tris(hydroxymethyl) aminomethane, AR, pH = 8.8) buffer, ethanol anhydrous (AR, 99.5%) and cupric sulphate anhydrous (CuSO_4_, AR) were purchased from Sinopharm Chemical Reagent Co., Ltd., Shanghai, China. Dopamine hydrochloride (DA, M_w_ = 189.64), hydrophobic fumed silica (SiO_2_ NPs, 7–40 nm, 100 m^2^·g^−1^) and anhydrous lithium chloride (LiCl, AR, 99.0%) were purchased from Macklin Biochemical Co., Ltd., Shanghai, China. All chemicals were used as received.

### 2.2. Preparation of the SBS Fibrous Membrane

**Figure 1:**
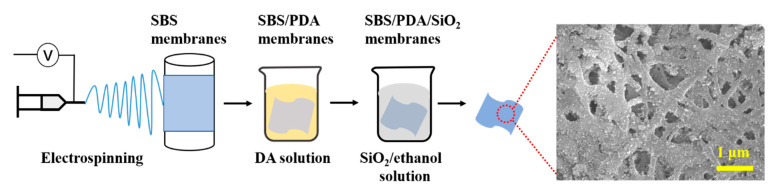
Schematic drawing showing the preparation of the SBS/PDA/SiO_2_ nanofibrous membrane and an SEM image of the prepared membrane.

A total of 9 g SBS was dissolved in a mixture of 68.25 g THF/22.75 g DMF (3/1, *w*/*w*) at room temperature with mechanical stirring for 4 h. Then, 0.004 g LiCl was added to the above mixture with continuous stirring for 30 min. The electrospinning solution with 9 wt% SBS and 0.004 wt% LiCl was obtained. All-in-one electrospinning equipment (ET-2535D, Beijing Ucalery Technology Development Co., Ltd., Beijing, China) was used in a horizontal setup, as shown in Figure 1. A total of 15 mL electrospinning solution was placed in a 20 mL syringe with a 20 G (internal diameter of 0.58 mm, external diameter of 0.91 mm) metallic needle. A 25 kV voltage was applied to the tip of the needle which kept a continuous jet stream. The flow rate of the microinjection pump was 0.5 mm∙min^−1^. The rotating rate of the rotating collector was 100 rpm. The distance between the needle and the collector was 100 mm. The temperature was kept. The electrospinning process was performed for 70 min under 27 ± 2 °C and humidity of 55 ± 2%. The obtained membrane was removed with solvent under 5 and 0.01 MPa for 12 h. Then, the SBS fibrous membrane was obtained.

### 2.3. Dip Coating of the SBS Fibrous Membrane with PDA and SiO_2_-NP

As shown in Figure 1, the post-processing consists of two steps: coating with polydopamine (PDA) and then SiO_2_ NPs. Dip coating with PDA: 5 mL of 1 M Tris solution was mixed with 495 mL deionized water, then 1 g of dopamine hydrochloride was added in the above solution, and the dopamine hydrochloride/tris solution with 2 g·L^−1^ dopamine hydrochloride was obtained. Then, the prepared SBS fibrous membrane was immersed in the above solution immediately and reacted for 4 h. Then, the obtained fibrous membrane was taken out and hung until no drips occurred. Finally, the SBS/PDA fibrous membrane was obtained.

Dip coating with SiO_2_ NPs: 8 g SiO_2_ NPs were dispersed in 392 g ethanol in a 500 mL beaker which was ultrasonically oscillated for 10 min followed by mechanical stirring for 30 min, and then the SiO_2_ NP/ethanol solution with 2.0 wt% SiO_2_ NPs was obtained. Then, the SBS/PDA fibrous membrane was immersed in the above solution for 1 h. Subsequently, the obtained fibrous membrane was dried at −0.01 MPa and 50 °C for 12 h. Finally, the SBS/PDA/SiO_2_ fibrous membrane was obtained. In the experiment, the SiO_2_ NPs/ethanol solutions with concentrations of 0.5 wt%, 1.0 wt%, 2.0 wt% and 4.0 wt% SiO_2_ NPs were adjusted.

### 2.4. Characterization of the SBS/PDA/SiO_2_ Fibrous Membrane

The surface morphology of the membranes was observed using SEM (JSM-IT300, JEOL Ltd., Tokyo, Japan). The pore size and distribution of the membrane were measured according to the bubble point method [47] with a capillary flow porometer (CFP-1500-AEXL, Porous Materials Inc., Ithaca, NY, USA).

The water contact angle (WCA) of the membrane was tested with a contact angle goniometer (DSA100, KRÜSS GmbH, Hamburg, Germany). The waterproof character of the membrane in terms of hydrostatic pressure (∆*P*) was tested according to the AATCC test method 127-2003 with a hydrostatic pressure tester (YG825E, Nantong Hongda Experiment Instruments Co., Ltd., Nantong, China). The increasing rate of water pressure was 6 kPa/min. The breathable performance of the membrane in terms of the water vapor transmission (WVT) rate was measured using the inverse cup water method according to ASTM E96/E96M-16 standard with a water vapor transmission tester (YG601H-II, Ningbo Textile Instruments Co., Ltd., Ningbo, China). The wind velocity was 1 m∙s^−1^. The temperature and relative humidity were 38 °C and 50%, respectively. The test time was 1 h. The WVT rate was calculated according to Equation (1) [45]:

(1) $$\mathrm{WVT}\;\mathrm{rate}=\frac{\Delta m}{S\times t}\times 24$$

where *S* is the testing area of the membrane, m^2^; *t* is the testing time, h; Δ*m* is mass change of the test cup during testing, kg. The windproof performance of the fibrous membrane in terms of air permeability was measured according to the ASTM D737-18 standard with an air permeability tester (YG461E/II, Ningbo Textile Instruments Co., Ltd., Ningbo, China). The pressure drop was 100 Pa. Honey, pure milk, juice, coffee solution (1 g coffee mixed with 30 mL water), black ink and CuSO_4_ particles were used as artificial contaminations to test the anti-fouling performance of the membrane [48,49].

## 3. Results and Discussion

**Figure 2:**
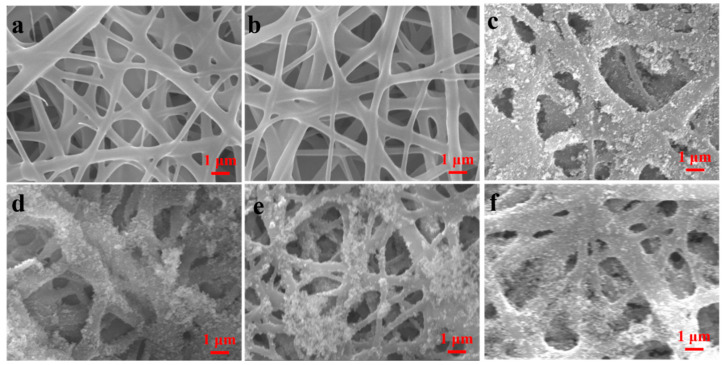
SEM images of (**a**) the SBS fibrous membrane, (**b**) the SBS/PDA fibrous membrane and (**c**)–(**f**) the SBS/PDA/SiO_2_ fibrous membrane with SiO_2_ NPs concentrations of (**c**) 0.5 wt%, (**d**) 1.0 wt%, (**e**) 2.0 wt% and (**f**) 4.0 wt%, respectively.

**Figure 3:**
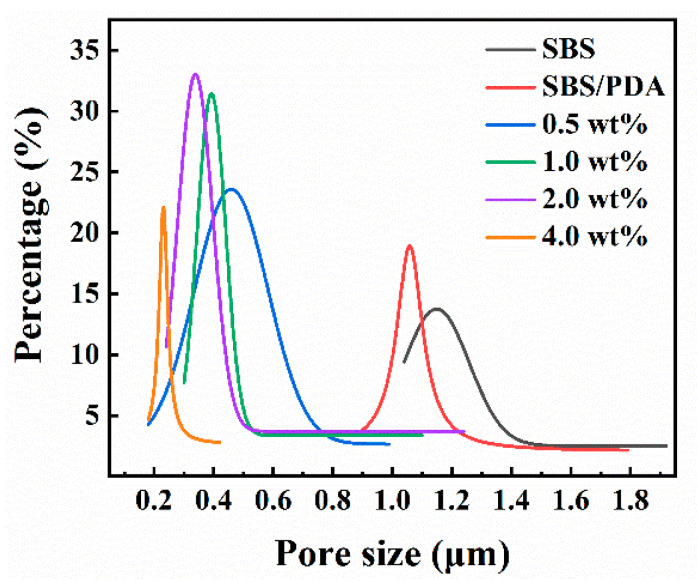
Pore size distribution of the SBS/PDA/SiO_2_ fibrous membranes with different SiO_2_ NPs concentrations.

The effect of concentration of SiO_2_ NPs on the morphology of the SBS/PDA/SiO_2_ fibrous membranes was evaluated. As shown in Figure 2a, the SBS fibers which have smooth surfaces were interwoven to form nonwoven webs. The SBS fibrous membrane has interconnected pore structures. The pore size of the membrane distributes within the range from 1.04 µm to 1.92 µm (Figure 3). The maximum pore size (*d_max_*) of 1.92 µm is the maximum value of pore size data. The mean pore size (*r*) is the average pore size. The *r* of the membrane is 1.44 µm (Table 1). After the in-situ polymerization of DA, the membrane morphology has little change. The pore size of the membrane distributes within the range from 0.90 µm to 1.86 µm (Figure 3). The *r* of the membrane slightly reduced to 1.34 µm (Table 1). As SiO_2_ NPs stick to surface of the SBS fibers by adhesive PDA, the SBS/PDA/SiO_2_ fiber forms a nano-scale convex character but retains pore structures (Figure 2c–f). The pore size of the SBS/PDA/SiO_2_ fibrous membrane with 0.5% SiO_2_ NPs decreases significantly compared with that of the SBS fibrous membrane (Figure 3). The *r* of the SBS/PDA/SiO_2_ fibrous membrane with 0.5% SiO_2_ NPs is 0.42 µm with a decline of 64% (Table 1). As the SiO_2_ NPs concentration increases from 0.5 wt% to 4 wt%, *d_max_* of the SBS/PDA/SiO_2_ fibrous membrane decreased from 1.02 μm to 0.45 μm, and *r* decreased from 0.42 µm to 0.30 µm, indicating that the pore size distribution of the SBS/PDA/SiO_2_ fibrous membranes becomes narrow and pore size declines, which is due to the significant SiO_2_ NPs coating on the surfaces of the SBS fibers.

**Figure 4:**
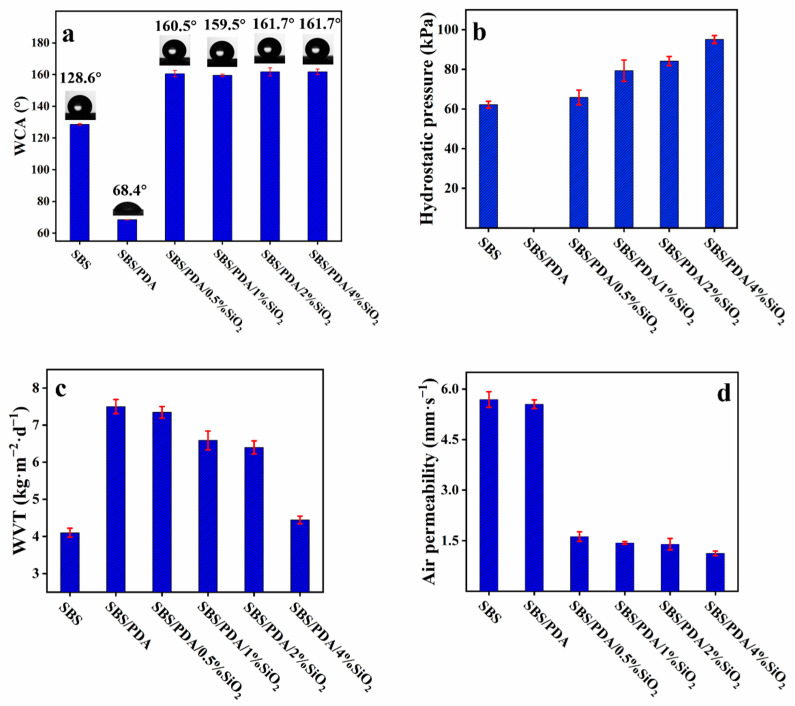
Properties of the fibrous membranes (**a**) WCA, (**b**) hydrostatic pressure, (**c**) WVT rate and (**d**) air permeability.

**Figure 5:**
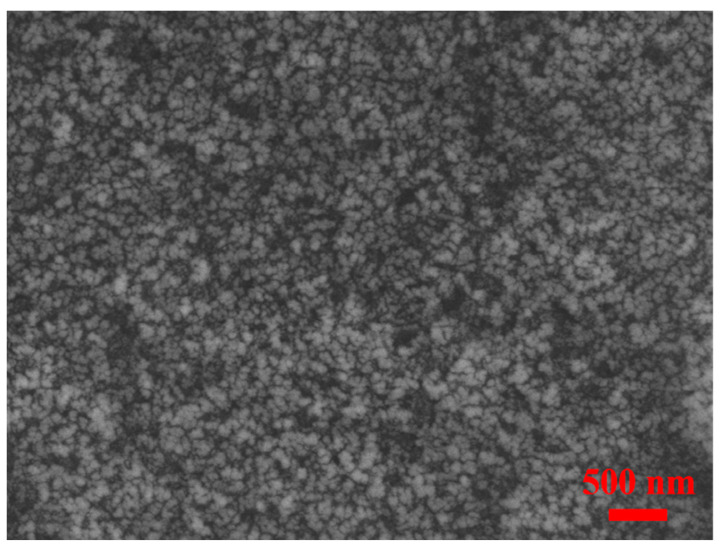
Image of the micro-convex composed of hydrophobic nanoscale SiO_2_ NPs.

The waterproof and breathable performance of the SBS/PDA/SiO_2_ fibrous membrane depends on its morphology and porous structure. In detail, as shown in Figure 4a, the WCA of the SBS fibrous membrane is 128.5°, which has good hydrophobicity because of the intrinsic hydrophobic SBS [45]. After in situ polymerization of DA, the WCA of the SBS/PDA fibrous membrane decreases sharply to 68°. One of the most important properties of PDA is its robust and strong adhesion to virtually all types of surfaces, regardless of the substrate’s chemistry [41,50]. Thus, PDA has opened a new route to the modification of various substrates [50]. The other is that the PDA displays high hydrophilicity because of the presence of many functional groups such as carboxy, amino, imine and phenol groups [50]. So, after the SBS fibers were coated with the PDA, the surface of the fibrous membrane becomes hydrophilic. In the next step, as SiO_2_ NPs adhere to the fibers by the adhesive PDA, the WCA reaches 160.5°, 159.5°, 161.7° and 161.7° with 0.5 wt%, 1 wt%, 2 wt% and 4 wt% SiO_2_ NPs, respectively, indicating that super-hydrophobic surfaces are obtained. It is inspired by the lotus leaf, which has a super-hydrophobic surface that is made up of papillose epidermal cells and an additional layer of epicuticular waxes [38]. Thus, the contact area is limited to the tips of the epicuticular wax crystals and the papillose epidermal cells when the liquid droplets or particles contact the surface of the lotus leaf [38]. So, the super-hydrophobic property of the SBS/PDA/SiO_2_ fibrous membrane can be attributed to the formation of the micro-convex structure with the coating of hydrophobic SiO_2_ NPs on the fiber surfaces, as shown in Figure 5. The micro-convex is composed of hydrophobic nanoscale SiO_2_ NPs, which enhances the effect of surface chemistry into super-hydrophobicity and reduces the contact area between water droplets and the surface of the fibrous membrane, simultaneously reducing droplet adhesion [51], which makes the fibrous membrane have a super water-repelling property.

Theoretically, ∆*P*(Pa) of the fibrous membrane is described by the Young–Laplace equation [6]:

(2) $$∆P=-\frac{4\gamma_{LG}cos\theta }{d_{max}}$$

where *γ_LG_* is the surface tension of water, N∙m^−1^; *θ* is water contact angle of the capillary wall in the membrane, which is dependent on the hydrophobicity of the membrane; *d_max_* is the diameter of the maximum pore of the porous membrane, m. The waterproof performance of a porous membrane depends on the wettability of the membrane and the parameters of *d_max_*. As shown in Figure 4b, the hydrostatic pressure of the SBS fibrous membrane is 62.2 kPa. It demonstrates that the SBS fibrous membrane is highly water resistant because the SBS has intrinsic hydrophobicity, while the *d_max_* of the membrane is at the microscale level. After coating with PDA, the hydrostatic pressure of the SBS/PDA fibrous membrane is below the detection limit, indicating that the SBS/PDA fibrous membrane has a poor water resistance because the PDA coating changes the membrane from hydrophobicity to hydrophilicity. As SiO_2_ NPs adhered to the fibers by PDA, the hydrostatic pressure of the SBS/PDA/SiO_2_ fibrous membrane increases obviously because the hydrophilic PDA coating is replaced with the hydrophobic SiO_2_ NPs coating. When the concentration of SiO_2_ NPs is 0.5 wt%, the hydrostatic pressure of the SBS/PDA/SiO_2_ fibrous membrane is 65.8 kPa after an increase of 5.9% compared with that of the SBS fibrous membrane. The hydrostatic pressure of the SBS/PDA/SiO_2_ fibrous membrane increases with increasing concentration of SiO_2_ NPs. When concentration of SiO_2_ NPs is 4.0 wt%, the hydrostatic pressure of the SBS/PDA/SiO_2_ fibrous membrane reaches a maximum of 95.1 kPa after an increase of 52.9% compared with that of the SBS fibrous membrane. In recent years, most of the reported contact angles of the fluorine-free fibrous membranes were below 160°, and the hydrostatic pressure was between 28.3 kPa and 106.2 kPa, as shown in Table S1 [4,36,37,52,53,54,55,56]. Thus, the SBS/PDA/SiO_2_ fibrous membrane has excellent waterproof properties.

The term “breathable” is used to define membranes that have moisture permeability in excess of 400 g·m^−2^·d^−1^ [11]. The breathable performance of the fibrous membrane is described by the Hagen SiO_2_ NPs Poiseuille equation [11,57]:

(3) $$Q=C\frac{Nr^{4}}{kb}∆P$$

where *Q* is the volume flux, (kg∙m^−2^∙s^−1^); *r* is the pore size, m; *N* is the porosity, equal to the pore numbers per unit area, m^−2^; *b* is the thickness of the fibrous membrane, m; *k* is the tortuosity of the pores; ∆*p* is the pressure drop across the fibrous membrane, Pa; and *C* is a constant. The breathable performance of a porous membrane depends on the parameters of *r*. The breathable performance was evaluated by measuring the WVT rate of a fibrous membrane. As shown in Figure 4c, compared with the SBS fibrous membrane, the WVT rate of the SBS/PDA fibrous membrane increases dramatically to 7.5 kg·m^−2^·d^−1^ after an 82.9% increase. The moisture permeability of the fibrous membrane has been significantly improved. From Table 1, the *r* of the SBS/PDA fibrous membrane is similar to the SBS fibrous membrane. According to the literature [12], the increasing moisture permeability should ascribe to the hydrophilic property of the PDA coating, which plays the role of “adsorption-diffusion-desorption” during the permeation of water vapor. At a 0.5 wt% SiO_2_ NPs coating on the fibers, the WVT rate of the SBS/PDA/SiO_2_ fibrous membrane slightly decreases to 7.3 kg·m^−2^·d^−1^ due to significant decreases in the *r*. It also can be found that the WVT rate of the SBS/PDA/SiO_2_ fibrous membrane decreases sharply when the concentration of SiO_2_ NPs is above 2 wt% because the *r* of the membrane decreases largely. Meanwhile, it is presumed that the PDA coating is completely covered by SiO_2_ NPs, which becomes an obstacle to the “adsorption-diffusion-desorption” of moisture. In recent years, most of the reported moisture permeability of fluorine-free fibrous membranes were between 0.719 kg·m^−2^·d^−1^ and 11.2 kg·m^−2^·d^−1^, as shown in Table S1 [4,36,37,52,53,54,55,56]. Thus, the moisture permeability of the SBS/PDA/SiO_2_ fibrous membrane is above average. Considering the wearing comfort, the concentration of the SiO_2_ NPs should be controlled below 2 wt%, and the moisture permeability of the SBS/PDA/SiO_2_ fibrous membrane above 6.4 kg·m^−2^·d^−1^, to obtain better moisture permeability of the fibrous membrane.

The air permeability of the fibrous membrane was tested to evaluate its windproof performance of the fibrous membrane. As shown in Figure 4d, the air permeability of the SBS fibrous membrane is 5.69 mm·s^−1^. By comparison, the air permeability of the SBS/PDA fibrous membrane has not obviously decreased. However, with the SiO_2_ NPs coating on the fibers, the SBS/PDA/SiO_2_ fibrous membrane shows dramatically declined air permeability, which is distributed in 1.1–1.6 mm·s^−1^, and it decreases slightly with increasing SiO_2_ NPs concentration. Compared with the SBS fibrous membrane and the SBS/PDA fibrous membrane, the *d_max_* and *r* of the SBS/PDA/SiO_2_ fibrous membrane decrease significantly, which restricts airflow. Indeed, the “adsorption-diffusion-desorption” effect of the PDA coating does not help air permeability. Thus, the windproof performance of the SBS/PDA/SiO_2_ fibrous membrane has improved greatly.

**Figure 6:**
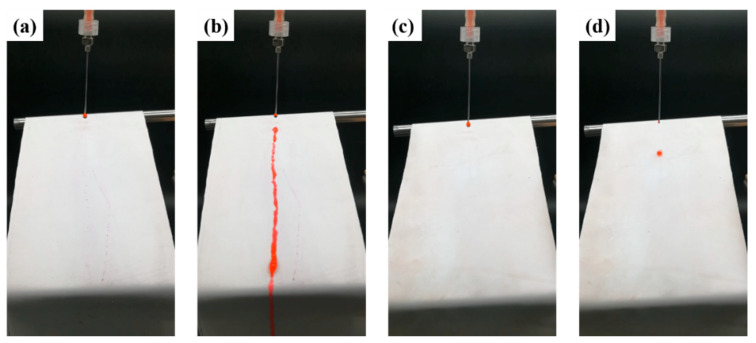
Images that demonstrate the super-hydrophobic property of fibrous membranes, (**a**,**c**) are the initial states of the SBS and the SBS/PDA/SiO_2_ fibrous membranes, (**b**,**d**) are the final states as the red dye droplets were dripped continuously on the surface of the SBS and the SBS/PDA/SiO_2_ fibrous membranes.

The super-hydrophobic property of the SBS/PDA/SiO_2_ fibrous membrane is demonstrated in Figure 6 and Movies S1 and S2. The fibrous membranes are placed on the shelf with an inclination angle of 25°. The red dye droplets are continually dripped on the surface of fibrous membranes. As shown in Figure 6a, b and Movie S1, the red dye droplets have an obvious wetting effect on the SBS fibrous membrane, which stick on the membrane surface, until a large droplet gathers, then falls with gravity, and leaves a line of red stain. Compared with the SBS fibrous membrane, the red dye droplets roll off immediately when they are dripped on the surface of the SBS/PDA/SiO_2_ membrane and leave no stain, as shown in Figure 6c, d and Movie S2.

**Figure 7:**
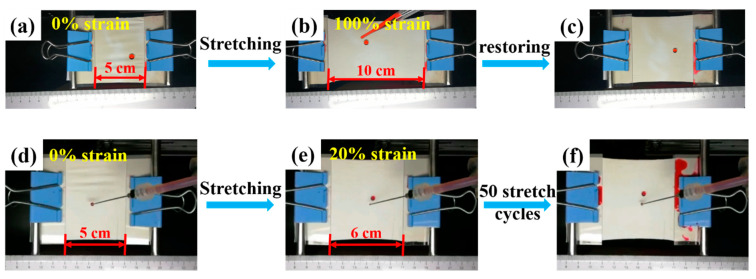
Hydrophobicity of the SBS/PDA/SiO_2_ fibrous membrane during dynamic tensile deformations with (**a**) 0% strain, (**b**) 100% strain, and (**c**) 0% strain in a cycle and (**d**) 0% strain and (**e**) 20% strain in a cycle and (**f**) after 50 stretch cycles of 0–20% strain.

**Figure 8:**
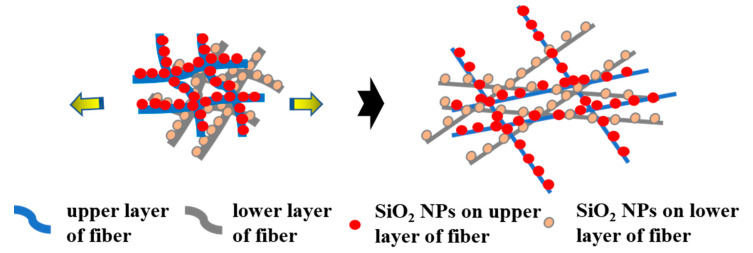
The schematic of the SBS/PDA/SiO_2_ fibrous membrane before and after stretching.

During the processing and wearing of textiles, deformation is very common and unavoidable. Thus, sustainable and stable properties of a functional textile appear to be particularly critical. According to our previous work [45], the SBS fibrous membrane showed excellent elastic properties. In this work, the super-hydrophobic property of the SBS/PDA/SiO_2_ fibrous membrane during dynamic tensile deformation was in situ observed. The red dye droplets are dripped continually on the SBS/PDA/SiO_2_ fibrous membrane surface as the strain of the membrane varies from 0 to 100% and then comes back to 0. As shown in Figure 7a–c and Movie S3, it can be found that the red dye droplets roll off the membrane and leave no stain during the completed stretch and recovery process, indicating that the super-hydrophobic property of SBS/PDA/SiO_2_ fibrous membrane has excellent stability under large deformation. The fibers in an electrospun fibrous membrane are randomly arranged and have a multilayer structure. As shown in Figure 8, when the water droplet is dripped on the membrane surface, the droplet is in contact with the fiber’s upper layer. During the stretching process, the fibers are elongated, resulting in a decrease in the distribution density of SiO_2_ NPs and an increase in the pore size of the membrane. However, the fiber’s lower layer is uncovered and plays a supporting role. So, the super-hydrophobic property of the SBS/PDA/SiO_2_ fibrous membrane does not decline, which shows the sustained and stable water-repelling property under large deformation. Meanwhile, the red dye droplets are dripped continually on the SBS/PDA/SiO_2_ fibrous membrane surface as a 20% strain was applied on the membrane for 50 stretching cycles. As shown in Figure 7d–f and Movie S4, after 50 stretching cycles, the SBS/PDA/SiO_2_ fibrous membrane shows no stain and no distortion, indicating its stable water-repellent performance and excellent elastic recovery property. Since 20% strain is the limit for most of the fabrics [45], the test proves that the SBS/PDA/SiO_2_ fibrous membrane can maintain its outstanding hydrophobicity for normal daily activities and exercises.

**Figure 9:**
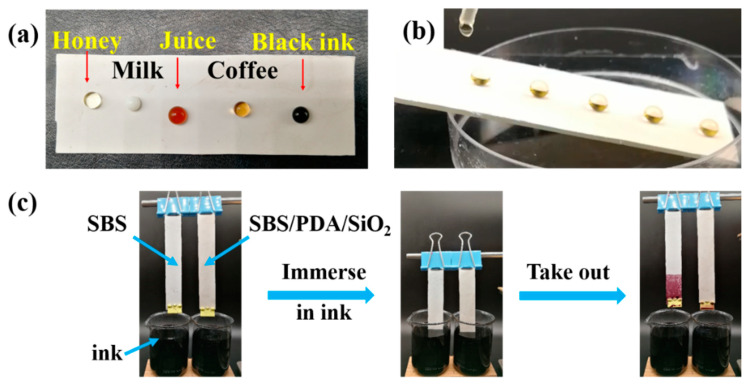
(**a**) Image of five kinds of droplets on the SBS/PDA/SiO_2_ fibrous membrane surface. (**b**) Image of honey drops rolling off the SBS/PDA/SiO_2_ fibrous membrane. (**c**) Images of the SBS fibrous membrane and the SBS/PDA/SiO_2_ fibrous membrane that were immersed in ink and taken out.

In addition to an aqueous solution of red dye, five common stains were chosen to further investigate the anti-fouling ability of the SBS/PDA/SiO_2_ fibrous membrane. As shown in Figure 9a, the droplets of honey, milk, juice, coffee and ink are dipped on the SBS/PDA/SiO_2_ fibrous membrane surface. Five droplets can maintain spherical shapes. Furthermore, as shown in Figure 9b and Movie S5, honey is dripped on the inclined SBS/PDA/SiO_2_ fibrous membrane, which rolls off the membrane and leaves no stain. Movie S5 also demonstrates the droplet tests of the other four types of liquids, which also roll off immediately with no traces left on the membrane surface. An additional experiment takes place in a cup of ink, as sown in Figure 9c. The SBS fibrous membrane and the SBS/PDA/SiO_2_ fibrous membrane are immersed in ink simultaneously. A few minutes later, the membranes are taken out from the ink. The results show that the SBS fibrous membrane is painted a deep purple–aubergine in the immersed part. In contrast, the SBS/PDA/SiO_2_ fibrous membrane has left no stain. These tests prove that the SBS/PDA/SiO_2_ fibrous membrane has excellent super-hydrophobic and anti-fouling properties.

**Figure 10:**
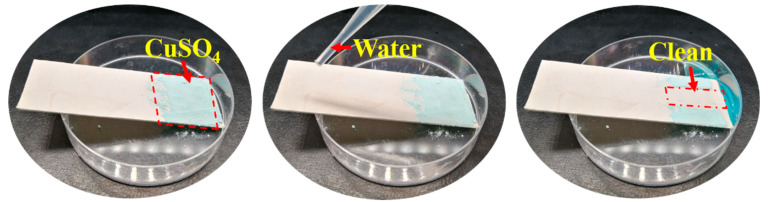
The SBS/PDA/SiO_2_ fibrous membrane contaminated with CuSO_4_ particles was washed cleanly with water.

As shown in Figure 10, a layer of CuSO_4_ particles is spread on the SBS/PDA/SiO_2_ membrane surface which is placed on an inclined plane. Then, the membrane surface is rinsed with a small amount of water. The CuSO_4_ particles in the place washed with water are carried away easily, leaving a clean surface. Due to the “lotus effect”, the dirt particles are picked up by water droplets due to the micro-convex architecture on the membrane surface, which minimizes the droplet’s adhesion to that surface. Thus, the SBS/PDA/SiO_2_ fibrous membrane exhibits excellent self-cleaning performance.

**Figure 11:**
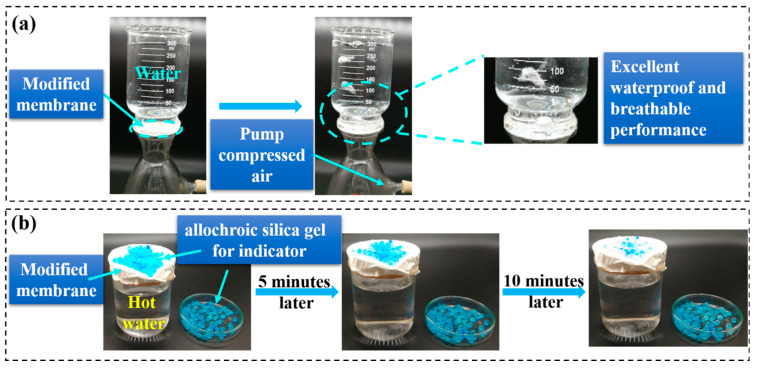
The waterproof and breathable performance of the SBS/PDA/SiO_2_ fibrous membrane. (**a**) Waterproof and breathable performance and (**b**) moisture permeability.

Two typical tests demonstrate that the SBS/PDA/SiO_2_ fibrous membrane possesses excellent waterproof and breathable performances. As shown in Figure 11a and Movie S6, the SBS/PDA/SiO_2_ fibrous membrane is placed on the sand core filter of a filter device. The above cup is filled with a 300 mL, 10.5 cm column of water. It can be found that there is no water permeating through the membrane under hydrostatic pressure, which can be ascribed to the super-hydrophobic surface and micron-pore structures of the fibrous membrane. Then, the air is pumped below bottle continuously. The air bubbles appear constantly in the above cup of water. The test reveals the excellent waterproof and breathable performance of the SBS/PDA/SiO_2_ fibrous membrane. Another test is shown in Figure 11b, the SBS/PDA/SiO_2_ fibrous membrane covers a beaker with 250 mL of 95 °C water. Then, the silica gel desiccants as moisture indicators are placed on the membrane surface and in a Petri dish at the same time. After 5 min, some of the silica gel desiccants on the membrane surface change from blue to colorless and transparent. However, there is no change in the silica gel desiccants in the Petri dish. After 10 min, most of the silica gel desiccants on the membrane surface turn to colorless while the silica gel desiccants in the Petri dish still have no change. This is further proof that the SBS/PDA/SiO_2_ fibrous membrane has outstanding moisture permeability.

**Figure 12:**
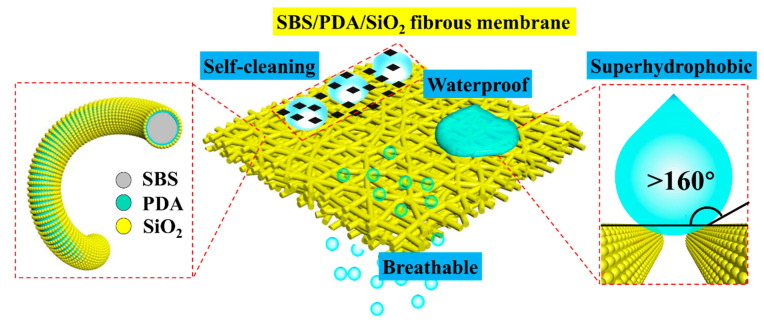
Schematic diagram of the SBS/PDA/SiO_2_ fibrous membrane.

In summary, as shown in Figure 12, the SBS/PDA/SiO_2_ fibrous membrane with micro–nano interconnected pores was fabricated using electrospinning technology. While inspired by the “Lotus effect” [51,58,59], the hydrophobic SiO_2_ NPs were adhered to SBS fibers with strong adhesive PDA to construct nano-convex structures. Consequently, the modified fibrous membrane which had a contact angle of above 160° and an outstanding elasticity exhibited stable super-hydrophobic properties under large and cyclic deformations. Meanwhile, the waterproof and breathable performance of the modified fibrous membrane was significantly improved.

**Figure 13:**
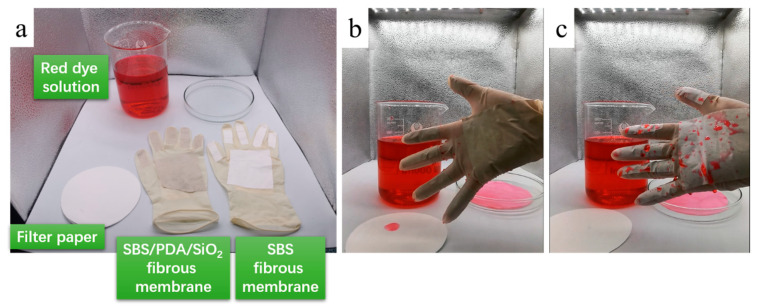
(**a**) The SBS and SBS/PDA/SiO_2_ fibrous membrane modified gloves, (**b**) the SBS/PDA/SiO_2_ fibrous membrane modified glove after immersion in a red dye solution, and (**c**) the SBS fibrous membrane modified glove after immersion in a red dye solution.

The high-performance fibrous membrane is suitable for people exposed to wastewater and pollution in the field of medicine and healthcare, paper and textile manufacturing, petroleum and the mining industry, as well as sewage services. In Figure 13a, the SBS and SBS/PDA/SiO_2_ fibrous membranes are attached on both sides of the fingers and palms of the latex gloves. In Movies S7 and S8, the experimenter wears the SBS and SBS/PDA/SiO_2_ fibrous membrane-modified gloves, respectively, and takes a piece of filter paper and immerses the filter paper and hand in a red dye solution. As a result, the filter paper is colored red rapidly. The uncovered surface of the glove turns slightly pink and leaves a dye stain. The surface of the SBS fibrous membrane drips with liquid beads of red dye (Figure 13b). Only the SBS/PDA/SiO_2_ fibrous membrane is undyed, and its surface has no drops of red dye remaining (Figure 13c). It can be expected that the fibrous membrane with excellent waterproof and breathable properties and self-cleaning performance ensures protective equipment, such as gloves, masks and suits, sit securely and remain comfortable even during prolonged periods of use, avoid troubles of wear and can be taken off frequently.

## 4. Conclusions

In conclusion, a fluorine-free, highly durable waterproof and breathable elastic nanofibrous membrane with self-clean performance was successfully obtained using a feasible and cost-effective electrospinning and dip coating process. Benefitting from the “lotus effect”, the SBS/PDA/SiO_2_ fibrous membrane with nanoscale convex surfaces by coating with hydrophobic SiO_2_ NPs was endowed with robust water repellency and excellent self-cleaning ability. In addition, the SBS/PDA/SiO_2_ fibrous membrane remained super-hydrophobicity under 100% strain and 20% strain cyclic loadings. When the concentration of SiO_2_ NPs was 4 wt%, the hydrostatic pressure of the modified fibrous membrane was 95.1 kPa ascribed to the micron scale *d_max_* and *r* of 0.45 µm and 0.30 µm, respectively, and super-hydrophobic surface with a contact angle of 161.7°. Attributed to the formation of the PDA interlayer, the moisture permeability of the SBS/PDA/SiO_2_ fibrous membrane with 0.5 wt% SiO_2_ NPs was enhanced with a WVT rate of 7.3 kg·m^−2^·d^−1^. However, as the concentration of the SiO_2_ NPs increased to 4 wt%, the WVT rate decreased to 4.4 kg·m^−2^·d^−1^, because the PDA coating was completely covered by a large amount of SiO_2_ NPs, and the *d_max_* and *r* of the membrane reduced to a minimum. Because of that, the windproof property of the SBS/PDA/SiO_2_ fibrous membrane was improved significantly. The air permeability of the modified fibrous membrane was 1.1 mm·s^−1^ when the concentration of the SiO_2_ NPs was 4 wt%. This work provided a simple and efficient modification method and designed a multi-functional fibrous membrane that has great potential in the manufacturing of personal protective equipment.

## Figures and Tables

**Table 1:** Pore sizes of the fibrous membrane.

Sample	*d_max_* (μm)	*r* (μm)
SBS	1.91	1.44
SBS/PDA	1.86	1.34
SBS/PDA/0.5%SiO_2_	1.02	0.42
SBS/PDA/1%SiO_2_	1.19	0.44
SBS/PDA/2%SiO_2_	1.04	0.39
SBS/PDA/4%SiO_2_	0.45	0.30

## Data Availability

Not applicable.

## Supplementary Materials

The following supporting information can be downloaded at: https://www.mdpi.com/article/10.3390/nano13030516/s1, Table S1: The reported data of the fluorine-free fibrous membranes in recent years; Movie S1: Wetting experiment on the surface of the SBS fibrous membrane; Movie S2: Wetting experiment on the surface of the SBS/PDA/SiO_2_ fibrous membrane; Movie S3: Wetting experiment on the surface of the SBS/PDA/SiO_2_ fibrous membrane during dynamic tensile deformations under 100% strain; Movie S4: Wetting experiment on the surface of the SBS/PDA/SiO_2_ fibrous membrane during dynamic tensile deformations under 20% strain and 50 stretching cycles; Movie S5: Droplet tests of various types of liquids on the surface of the SBS/PDA/SiO_2_ fibrous membrane; Movie S6: Waterproof and breathable performance of the SBS/PDA/SiO_2_ fibrous membrane; Movie S7: Anti-fouling experiment of the SBS fibrous membrane modified glove; Movie S8: Anti-fouling experiment of the SBS/PDA/SiO_2_ fibrous membrane modified glove.
