# Supplementary material for: Fluorine-Free, Highly Durable Waterproof and Breathable Fibrous Membrane with Self-Clean Performance
Source: Nanomaterials (Basel). 2023 Jan 27;13(3):516. doi: 10.3390/nano13030516 (PMC9922014; doi:10.3390/nano13030516)
Supplement: Supplementary file 1 [file nanomaterials-13-00516-s001.zip › nanomaterials-2153676-supplementary.pdf]

nanomaterials

Supplementary

Fluorine-Free, Highly Durable Waterproof and Breathable Fibrous Membrane with Self-Clean Performance

Jinchao Zhao [1], Teng Zhang [2], Youmu Li [2], Leping Huang [2,*] and Youhong Tang [3,*]

1 Hubei Provincial Engineering Laboratory for Clean Production and High Value Utilization of Bio-Based Textile Materials, Wuhan Textile University, Wuhan 430200, China
2 School of Material Science and Engineering, Wuhan Textile University, Wuhan 430200, China
3 Flinders Institute for NanoScale Science and Technology, College of Science and Engineering, Flinders University, Adelaide, SA 5042, Australia
* Correspondence: lphuang@wtu.edu.cn (L.H.); youhong.tang@flinders.edu.au (Y.T.)

Table S1. The properties of the fluorine-free fibrous membrane in recent literature.

Fluorine-free fibrous membrane	Contact angle (°)	Hydrostatic pressure (kPa)	Moisture permeability ($kg\cdot m^{-2}\cdot d^{-1}$)	Air permeability ($mm\cdot s^{-1}$)	Durability	Reference
Polyamide 6 / TiO_2 NPs	129.5	106.2	10.3	-	-	Zhao *et al.*, 2020
Waterborne polyurethane / polycarbodiimide/ long-chain alkyl polymer	137.1	35.9	4.885	19.9	-	Zhou *et al.*, 2021
Silicon-based polyurethane/polymethyl methacrylate/ hydrophobic octadecanethiol	131	64.43	7.879	-	-	Tian *et al.*, 2022
Polymers of intrinsic microporosity-3,3'-Dimethylbiphenyl-4,4'-diamine	above 130	88.7	11.2	20	-	Ren *et al.*, 2022
Polyamide / polydimethylsiloxane	134.1	28.3	3.77	12.7	-	Zhou *et al.*, 2022
Polyacrylonitrile/ Amino functional modified polysiloxane	137.2	93.8	4.7	12.7	-	Zhang *et al.*, 2022
Polyurethane/ Siliceous polyurethane/stearic acid	133	79	8.0	-	-	Zhang *et al.*, 2022
Polyacrylonitrile/TiO_2 NP/ hexadecyltrimethoxysilane/polydimethylsiloxane	147	50	0.719	70.65	Hydrostatic pressure of 40 kPa After 30 times of washing	Gu *et al.*, 2023
SBS/PDA/SiO2 fibrous membrane	161.7	84.2	6.4	1.1	No deterioration after applying 20% strain for 50 stretching cycles	Current work

"-" means no data.
